# Procedural case: Ultrasound Case: Syncope in PE

**DOI:** 10.21980/J8.52365

**Published:** 2025-12-31

**Authors:** Neil Wallace, Andrew Melendez, Lars Beattie, Tina Chen, David Fernandez, Amrita Vempati, Kelly Roszczynialski, Stephanie Cohen, Stephanie Stapleton, Tiffany Moadel

**Affiliations:** 1 University of Arizona/Banner Medical Center, Department of Emergency Medicine, Phoenix, AZ; 2 Yale University, Department of Emergency Medicine, New Haven, CT; 3 University of Florida, Department of Emergency Medicine, Gainesville, FL; 4 St Louis University, Department of Emergency Medicine, St Louis, MO; 5 Mount Sinai Hospital, Department of Emergency Medicine, Brooklyn, NY; 6 Creighton School of Medicine Phoenix, Department of Emergency Medicine, Phoenix, AZ; 7 Stanford University, Department of Emergency Medicine, Palo Alto, CA; 8 University of Central Florida, Department of Emergency Medicine, Orlando, FL; 9 Boston University/Boston Medical Center, Department of Emergency Medicine, Boston, MA; 10 Zucker School of Medicine at Hofstra/Northwell, Department of Emergency Medicine, Hempstead, NY

## Abstract

**Audience:**

This certifying exam practice ultrasound case is intended for emergency physicians (EP) in training.

**Introduction:**

Point-of-care ultrasound (POCUS) has become an essential tool in the practice of Emergency Medicine (EM). EM physicians routinely use POCUS to expedite diagnostic evaluations, guide resuscitative efforts, enhance the safety and success of bedside procedures, and reassess critically ill patients in real-time. Furthermore, POCUS is listed in the Model of the Clinical Practice of Emergency Medicine from ABEM, including diagnostic, resuscitative, and procedural components.^1^ Training during residency, however, remains variable while POCUS continues to grow in importance.^2^ Incorporating structured, competency-based ultrasound education into EM training has the potential to standardize skill with image acquisition, enhance image interpretation, improve confidence with clinical integration, and ultimately lead to better patient care in the acute setting.

**Educational Objectives:**

By the end of the session, learners will be able to: 1) obtain and interpret the parasternal short-axis view of the heart to assess right ventricular size and function, 2) identify ultrasound findings suggestive of pulmonary embolism (PE) on cardiac short-axis view, including right ventricular dilation and septal bowing, 3) demonstrate appropriate probe selection and positioning to obtain optimal images of the heart and inferior vena cava (IVC), 4) evaluate the IVC using a subxiphoid or longitudinal view to assess distension and lack of respiratory collapse as supportive findings for elevated right heart pressures, 5) identify the anatomy of the neck vasculature, differentiate between the internal jugular vein and carotid artery, and select the appropriate puncture site, 6) describe ultrasound-guided central venous catheterization via the right internal jugular vein, using a sterile technique and real-time guidance.

**Educational Methods:**

We developed a single-station Objective Structured Clinical Examination (OSCE) focused on point-of-care ultrasound (POCUS) in the evaluation of a patient with syncope and suspected pulmonary embolism (PE).^3^ This format aligns with the American Board of Emergency Medicine’s (ABEM) newly implemented certification examination, emphasizing real-time clinical reasoning and ultrasound skills in a simulated encounter.^4^ The OSCE features a simulated participant and a standardized examiner script mirroring the ABEM certification exam format to ensure realism and consistency.^5^,^6^ ABEM has not released specific scoring elements to our knowledge. As a result, a novel set of grading criteria was developed based on patient-centered care, image acquisition, and image interpretation. The case was co-developed by experts in emergency ultrasound and simulation-based education, and then peer-reviewed to ensure clinical accuracy, clarity, and educational value.

During the station, the examinee is presented with a focused clinical case involving a patient with syncope and is expected to demonstrate foundational POCUS knowledge, including image acquisition, optimization, and interpretation to assess for signs of PE, such as right ventricular strain or plethoric inferior vena cava.^7^ The examiner follows a structured and standardized script that evaluates the participant using a checklist, including de-identified images and clips of pathology. This tool assesses image acquisition, interpretation, and the integration of ultrasound findings into clinical decision-making. This OSCE provides a standardized method to assess diagnostic and procedural competence in a common application of ultrasound.

**Research Methods:**

This simulation case can be used as a standalone scenario or part of an ABEM Certifying Exam practice. For best results, residents should complete it individually, with some assigned as facilitators to better understand the examiner’s role. The case runs for about 10 minutes, followed by a 10-minute debrief. Optimal setup includes examiner control over room lighting and ultrasound display. The case was trialed with EM residents across multiple sites using an iterative process. Feedback was collected via anonymous Qualtrics surveys using Likert scales and open-ended comments. The Boston University Institutional Review Board reviewed the project and deemed it exempt.

**Results:**

Initial testing with two residents and a facilitator showed the case was clear, easy to use, and valuable for exam prep. A second round at the 2025 SAEM Annual Meeting included four learners and one facilitator. Feedback led to case revisions. Survey scores were consistently positive, with high ratings for clarity, usability, and relevance to ABEM exam preparation.

**Discussion:**

The development and implementation of a syncope-focused ultrasound case for ABEM certification preparation highlights the growing integration of POCUS into emergency medicine training and assessment. Syncope is a high-yield chief complaint in the emergency department and serves as a common clinical context in which critical diagnoses like PE may be encountered.^8^ In this case, the use of bedside ultrasound to identify right heart strain consistent with PE not only mirrors real-world clinical practice but also reinforces the diagnostic reasoning and image interpretation skills essential for contemporary emergency physicians.^9^,^10^ The favorable reception from learners underscores the value of ultrasound-based cases in exam preparation and supports the continued development of high-quality, board-style ultrasound assessments in emergency medicine training programs.

**Topics:**

Ultrasound, cardiac, inferior vena cava, vascular, pulmonary embolism.

## USER GUIDE

**Table:** 

List of Resources:	
Abstract	126
User Guide	129
For Examiner Only	132
Certifying Exam Assessment	137
Stimulus	140
Debriefing and Evaluation Pearls	146

**Learner Audience:**

This case is appropriate for interns and junior and senior residents.

**Time Required for Implementation:**

Case: 5–10 minutes

Debriefing: 5 minutes

**Recommended number of learners per instructor:**

All Emergency Medicine physicians in training

**Topics:**

Ultrasound, cardiac, inferior vena cava, vascular, pulmonary embolism.

**Objectives:**

By the end of the session, learners will be able to:

1. Obtain and interpret the parasternal short-axis view of the heart to assess right ventricular size and function.
2. Identify ultrasound findings suggestive of PE on cardiac short-axis view, including right ventricular dilation and septal bowing.
3. Demonstrate appropriate probe selection and positioning to obtain optimal images of the heart and IVC.
4. Evaluate the IVC using a subxiphoid or longitudinal view to assess distension and lack of respiratory collapse as supportive findings for elevated right heart pressures.
5. Identify the anatomy of the neck vasculature, differentiate between the internal jugular vein and carotid artery, and select the appropriate puncture site.
6. Identify the location of a central line guidewire within the internal jugular vein.

### Linked objectives, methods and results

This ultrasound case introduces a 28-year-old female who presents to the emergency department after a witnessed syncopal episode. The facilitator begins by providing the initial information about the patient and prompts the learner to ask them for any probe changes, changes in depth, gain, or ultrasound mode to assist them with the case, as well as if they need any help in repositioning the patient on the bed. The learner will then be asked to obtain and interpret a parasternal short-axis view of the heart (Objective 1). The learner will then be asked to interpret a video of abnormal pathology provided by the examiner (Objective 2). The learner will then be asked to obtain and interpret a subxiphoid view of the IVC (Objective 3). The learner is then expected to interpret a video of abnormal pathology in this view (Objective 4). The learner will then be asked to obtain a view of the right internal jugular vein for intravenous access (Objective 5). Lastly, the learner will be asked to interpret a video of the right internal jugular vein for intravenous access (Objective 6).

### Recommended pre-reading for instructor

1. Basic | Sonoguide
  - Prats M, Bahner D. Echocardiography for Emergency Physicians. ACEP Sonoguide. Published 2020. https://www.acep.org/sonoguide/basic/cardiac
  - Kotwal A, Bryczkowski C, Mirza M. Vascular Access. ACEP Sonoguide. Published August 18, 2020. Accessed November 21, 2025. https://www.acep.org/sonoguide/procedures/vascular-access
2. 5 Minute Sono - Core Ultrasound
  - Avila J. Central Venous Access (2020 edition). Core Ultrasound. Published July 15, 2020. Accessed November 21, 2025. https://coreultrasound.com/central-venous-access/
  - Avila J. Right Heart Strain. Core Ultrasound. Published June 28, 2021. Accessed September 25, 2023. https://coreultrasound.com/right-heart-strain/
  - Avila J. 5 Min Sono – IVC (2021). Core Ultrasound. Published August 18, 2021. https://coreultrasound.com/ivc/

### Results and tips for successful implementation

This simulation case can be implemented either as a standalone scenario or as part of a comprehensive ABEM Certifying Exam practice session. For optimal realism, we recommend that residents complete the case individually. Assigning learners to the facilitator role may enhance their understanding of the examiner’s approach. The case is intended to take approximately 10 minutes to complete, followed by a 10-minute debriefing for directed feedback. Whenever possible, the room should be set up to allow the examiner to control both the lights of the room and the ultrasound machine itself to optimize control of the examinees’ views. The examiner should also have preloaded stimuli on a separate device that are unlabeled for the examinee to review when prompted. We tested the case on four learners and one facilitator. We used an iterative case trialing process at multiple sites with a convenience sample of EM residents. Both the facilitators and residents provided experience feedback via anonymous surveys using Likert scales and open comment space. Likert scales ranged from 1 (strongly disagree) to 5 (strongly agree). All data were collected using Qualtrics (https://www.qualtrics.com) and analyzed using Excel (Microsoft, Redmond, WA). The Boston University Institutional Review Board reviewed the project and deemed it exempt.

During the first round of trialing, a facilitator at an alternate academic site tested the case with 2 EM residents. Facilitators completed a SSET survey to evaluate the quality of key simulation elements.^11^ Residents completed a modified usability survey. We performed a second round of case trialing at the Society for Academic Emergency Medicine Annual Meeting during May 2025 (Philadelphia, PA). One facilitator and four learners completed modified usability surveys. We modified the case based on survey feedback.

The SSET data were largely positive; case objectives, key actions, and materials were clear. The facilitator found the case was easy to use (4.0) and thought others would feel similarly (4.0). They felt confident using the case (4.0) and would like to use this case for ABEM certifying exam practice (5.0). Four learners found both the written and verbal case materials to be clear (4.5, 4.8). They found the experience was helpful practice for the ABEM certifying exam (4.0).

In addition to quantitative ratings, evaluators provided open-ended feedback on suggested improvements, helpful elements of the case, and clinical topics they would like to see in this format. A section was also included for any additional comments, where evaluators commented positively on the clarity of the case’s instructions and favored that the scenario was task focused.

One area of constructive feedback centered on probe placement and image acquisition. While the case was designed around a parasternal short axis (PSAX) view of the heart, several examinees expressed a preference for alternative acoustic windows, such as the apical four-chamber view, to evaluate right heart strain. This variability in preferred scanning approach highlights both the flexibility of POCUS and the importance of allowing examinees some degree of autonomy in image acquisition to better reflect the diversity of real-life practice patterns. Future iterations of similar educational tools may benefit from incorporating branching logic or allowing multiple ultrasound views to better assess learners’ clinical adaptability and probe technique.

Overall, this case demonstrates that simulation-based ultrasound scenarios can effectively mirror the known elements of the ABEM exam while providing a practical, meaningful learning experience.

## Figures and Tables

**Table:** 

Patient-Centered Care	Yes	No
Introduce yourself to the patient and wear gloves.		
Explain to the patient they will be using the ultrasound to assess their heart and parts of their blood vessels.		
Warn the patient of pressure when applying the probe for cardiac ultrasound.		
**Image Acquisition – (Parasternal Short-Axis View of Heart)**		
Probe - selects appropriate probe **- phased array probe**		
Depth - appropriately set depth or appropriate depth for visible anatomy **- visualized descending aorta or 1 cm beyond the pericardium**		
Gain - appropriate set gain or appropriate for visible anatomy **- can visualize the endocardial border, or appropriate to visualize left ventricular contraction**		
Quality - basic anatomy is visible and identifiable to the examiner **- left ventricle and right ventricle in cross-section**		
**Image Interpretation – (Parasternal Short-Axis View of Heart)**		
Anatomy - Appropriately identifies the **left ventricle** (basic anatomy)		
Anatomy - Appropriately identifies the **right ventricle** (basic anatomy)		
Advanced Anatomy - Appropriately identifies the **pericardium** (advanced anatomy)		
Application - Appropriately identifies **either D-sign or septal flattening**		
Application - Appropriately identifies **right ventricular enlargement**		
**Image Acquisition – (Subxiphoid View of IVC)**		
Probe - selects appropriate probe - **phased array or curvilinear probe**		
Depth - appropriately set depth or appropriate depth for visible anatomy, **1–2 cm beyond the IVC**		
Gain - an appropriate set gain or appropriate for visible anatomy **- can visualize the walls of the IVC**		
Quality - basic anatomy is visible and identifiable to the examiner - **can visualize the IVC and the junction of the IVC/right atrium, IVC and hepatic veins, or the IVC/diaphragm interface**		
**Image Interpretation – (Subxiphoid View of IVC)**		
Anatomy - Appropriately identifies **liver** (basic anatomy)		
Anatomy - Appropriately identifies **IVC** (basic anatomy)		
Advanced Anatomy - Appropriately identifies **either the IVC junction with the right atrium OR the hepatic veins draining into the IVC, OR the IVC/diaphragmatic junction **(advanced anatomy)		
Application - Appropriately identifies **dilated IVC or IVC with minimal respiratory variation**		
Application - Appropriately identifies **this constellation of findings suggesting right heart strain** OR **right heart dysfunction**		
**Image Acquisition – (Right Internal Jugular Vein)**		
Probe - selects appropriate probe - **linear probe**		
Depth - appropriately sets depth or appropriate depth for visible anatomy--**can visualize the superficial and deep walls of the carotid artery and internal jugular vein**		
Gain - an appropriate set gain or appropriate for visible anatomy--**can visualize the walls of the internal jugular vein**		
Quality - basic anatomy is visible and identifiable to the examiner--**can visualize both the carotid artery and internal jugular vein**		
**Image Interpretation – (Right Internal Jugular Vein)**		
Anatomy - Appropriately identifies the **internal jugular vein** (basic anatomy)		
Anatomy - Appropriately identifies the **carotid artery** (basic anatomy)		
Application - Appropriately identifies the **guidewire in the internal jugular vein**		
Advanced Anatomy - Appropriately identifies **reverberation and/or comet tail artifact from the guidewire** (ultrasound artifact)		

**Table:** 

**Candidate Task Sheet**	
**#1**	**D-sign US**
**#2**	**Plethoric IVC US**
**#3**	**Short axis guidewire and long axis guidewire US**

**Table:** Ultrasound Candidate Task Sheet

**CASE PARAMETERS**
This is a (5–10) minute ultrasound case. You will acquire 2–3 ultrasound images and interpret 2–3 ultrasound videos. Please know that the examiner may interrupt you to move through the case in a timely manner. **Expectations for Standardized Patient Interactions** Upon entering the room, the examiner will introduce the patient. You should interact with the standardized patient as you would any patient in a clinical situation, but please: Wear gloves. Ask the patient to expose parts of their body as needed. Do not wipe the gel off the patient; they will remove gel themselves. Ask the patient to reposition themselves to facilitate image acquisition as needed. Adjust the bed as needed or ask the examiner. Do not acquire any further history in your assessment of this patient. **Expectations for Examinee Interactions** You must verbalize your thoughts while you are performing ultrasounds. Once you obtain a representative image, ask the examiner to “freeze” the screen. **Expectations for Ultrasound Machine Manipulation** You will manipulate the ultrasound probe; the examiner will operate the machine Ask the examiner to adjust machine settings (depth, gain, or other modes) or transducer probe selection (linear, curvilinear, or phased array) as necessary. Notify your examiner if you would like to make any measurements.
**PATIENT INFORMATION**
A 28-year-old female with no past medical history presents after a syncopal episode at home. She reports worsening shortness of breath and chest discomfort over the last few days.
**PHYSICAL EXAM FINDINGS**
Vital Signs: BP 89/57, HR 128, RR 24, T 98.4° F (36.9° C), SpO2 89% on RA
**TASK STATEMENT**
Your tasks are as follows: Obtain a parasternal short-axis view of the heart. Interpret a video of a parasternal short-axis view of the heart. Obtain a subxiphoid view of the inferior vena cava (IVC). Interpret a video of the inferior vena cava (IVC). Obtain a view of the right internal jugular vein for intravenous access. Interpret a video of the right internal jugular vein for intravenous access.

**STIMULUS 1 f1-jetem-10-5-ce126:**
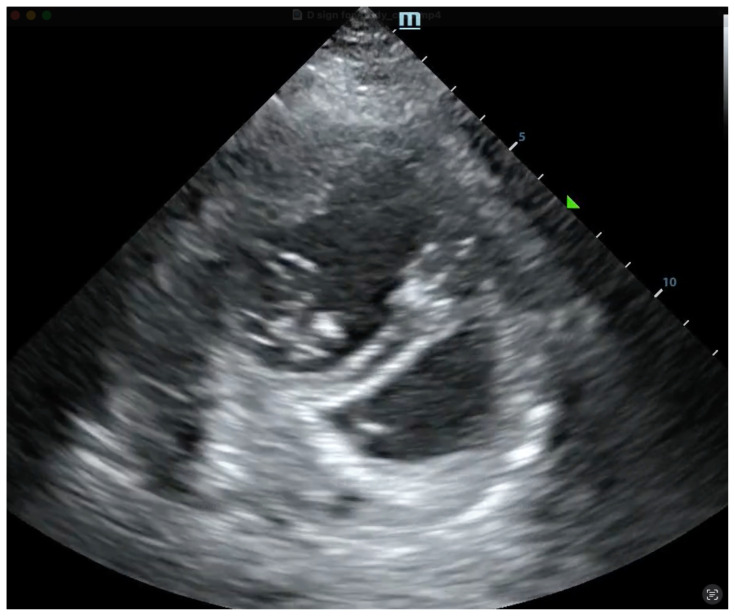
Video Links: https://youtu.be/Fmy8x-ve0So Author’s own image.

**STIMULUS 2:**
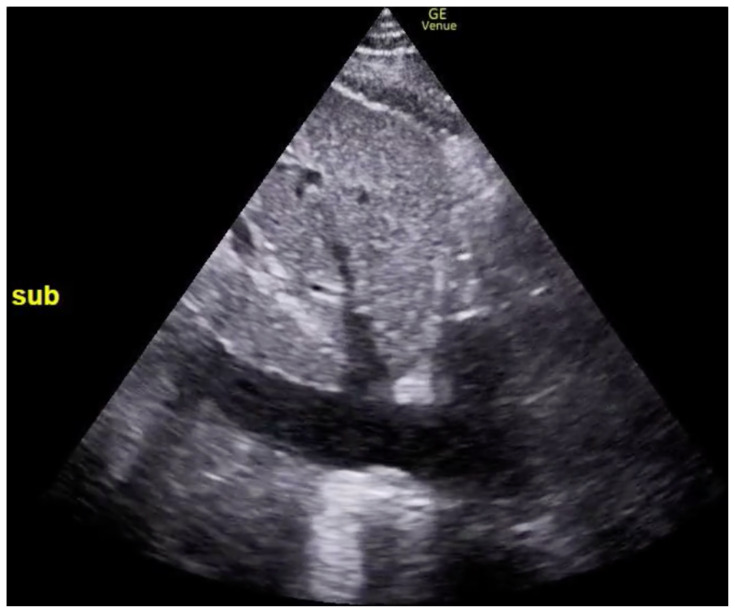
Video Links: https://youtu.be/pWdmA0wSN_w Author’s own image.

**STIMULUS 3:**
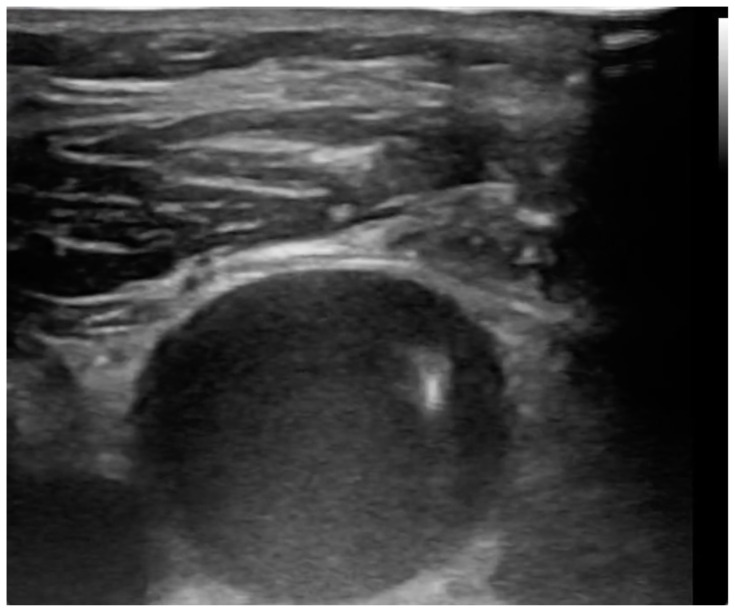
Video Links: https://youtu.be/pdqBEDS7wmQ Author’s own image.

**STIMULUS 3:**
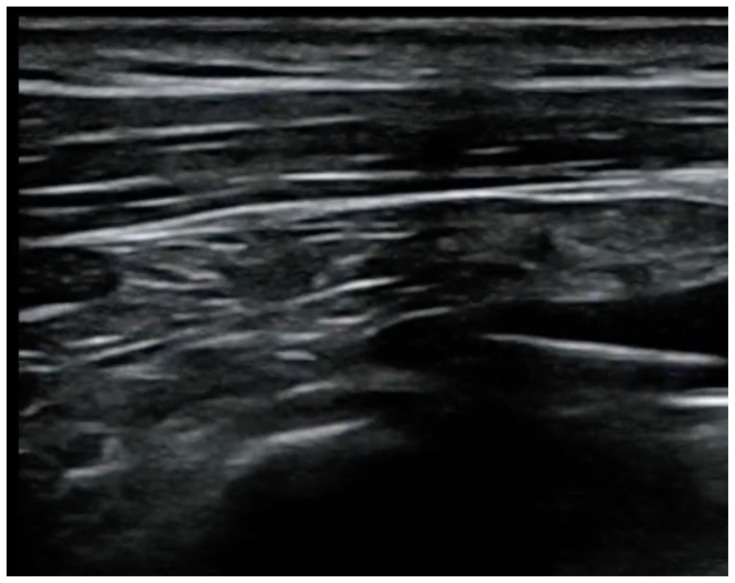
Video Links: https://youtu.be/oe1tuAiKLWQ Author’s own image.

## FOR EXAMINER ONLY

### Ultrasound Case: Syncope in PE Summary

**Diagnosis:** Pulmonary Embolism

**Case Summary:** This ultrasound case involves a 28-year-old female who presents to the emergency department after a witnessed syncopal episode at home. The facilitator guides the learner through a series of prompts to elicit ultrasound acquisition skills to make clinical decisions and interventions. The patient can provide her history, including shortness of breath and chest discomfort over the last few days. The physical exam reveals signs concerning for a massive pulmonary embolism (PE), including hypotension, tachycardia, and hypoxia. As the case proceeds, it is determined that the patient will require central access through a right internal jugular (IJ) central line. The learner is expected to utilize ultrasound to refine the differential diagnosis for syncope. Key ultrasound findings include septal bowing in short-axis view, a plethoric IVC without respiratory variability, identification of the internal jugular vein, and identification of a central line guidewire within the internal jugular vein. Throughout the scenario, the learner is asked to identify normal anatomy on a standardized participant and to identify abnormal findings on visual stimuli provided by the examiner.

**Standardized Actor Profile**:

*Patient demographics:* female adult, recommended to be young to middle-aged given the clinical vignette; however, given the limitations of standardized actors, age may be abstained as a consideration.

*Profile:* The patient’s motivation is to understand why she lost consciousness. She has been feeling progressively short of breath for the past few days, with some associated mild chest pain. Because she is short of breath, she responds in shorter sentences (in the range of 6–10 words, before pausing to take a breath). She is colloquially familiar with ultrasound for pregnancies but has never had one performed on her heart or other parts of her body. She is accepting of answers provided by the physician, without significant further probing questions. The purpose of this examination is primarily to assess clinical ultrasound skills and knowledge with very limited assessment of doctor-patient interaction.

**Materials/personnel needed:**

1 Instructor

1 Standardized participant

1 Ultrasound machine

1 Bottle of ultrasound gel and/or 2–3 individual ultrasound gel packets

**Room Setup:**

Stretcher for a standardized participant

### Ultrasound Case: Syncope in PE Examiner Script

**Examiner Script:**

“Hello, Doctor. I am Dr. _ and will be assisting as your examiner for this ultrasound case. As a reminder, please ask me to change probes, change depth, gain, or mode of ultrasound to assist you with the case. Please ask for help to reposition the patient on the bed. Do you have any questions?”

**Case vignette:**

“As you have noted, the patient is a 28-year-old female with no past medical history who presents after a syncopal episode at home. She reports worsening shortness of breath and chest discomfort over the last few days.”

**For diagnostic ultrasound:**

“I would like for you to demonstrate how you would assess syncope using ultrasound to evaluate for right ventricular strain. Which probe would you like to use?”

**Probe settings:**

- Linear: Gain 10, depth 5 cm
- Phased array: Gain 10, depth 2 cm
- Curvilinear: Gain 10, depth 2 cm

**If asked to change probe settings:**

“Tell me when to stop adjusting (gain/depth).”

Adjust the gain and depth slowly, allowing the examinee to stop you.

“When you find an acceptable image, please let me know.”

***Learner Response***

**After the examinee has selected their image:**

“Thank you, I will take the probe from you now. Please direct your attention to the screen. Assume this video is the video you obtained from this patient. I would like you to point out any artifacts, anatomy, or pathology that would be important for this complaint.”

#### Show Stimulus 1

**For diagnostic ultrasound:**

“I would like for you to now demonstrate how you would assess syncope using ultrasound to evaluate the patient’s inferior vena cava for collapsibility. Which probe would you like to use?”

**Probe settings:**

- Linear: Gain 10, depth 5 cm
- Phased array: Gain 10, depth 2 cm
- Curvilinear: Gain 10, depth 2 cm

**If asked to change probe settings:**

“Tell me when to stop adjusting (gain/depth).”

Adjust the gain and depth slowly, allowing the examinee to stop you.

“When you find an acceptable image, please let me know.”

***Learner Response***

**After the examinee has selected their image:**

“Thank you, I will take the probe from you now. Please direct your attention to the screen. Assume this video is the video you obtained from this patient. I would like you to point out any artifacts, anatomy, or pathology that would be important for this complaint.”

#### Show Stimulus 2

**For procedural ultrasound:**

“Doctor, what I would like for you to do now is demonstrate how to use the ultrasound to perform central venous IV access on the right internal jugular vein. Which probe would you like to use?”

**Probe settings:**

- Linear: Gain 10, depth 5cm
- Phased array: Gain 10, depth 2cm (or as shallow as able)
- Curvilinear: Gain 10, depth 2cm (or as shallow as able)

**If asked to change probe settings:**

“Tell me when to stop adjusting (gain/depth).”

Adjust the gain and depth slowly, allowing the examinee to stop you.

“When you find an acceptable image, please let me know.”

***Learner Respons*** *e*

**After the examinee has selected their image**:

“Thank you, I will take the probe from you now. Please direct your attention to the screen. Assume this video is the video you obtained from this patient. I would like you to point out any artifacts, anatomy, or pathology that would be important for this procedure.”

#### Show Stimulus 3

**End of case:**

“Thank you, that concludes your case.”

## CERTIFYING EXAM ASSESSMENT

*Ultrasound Case: Syncope in PE*

Learner: _________________________________________

**Summative and formative comments:**

### Stimulus Inventory

[Fig f1-jetem-10-5-ce126]

## DEBRIEFING AND EVALUATION PEARLS

### Ultrasound Case: Syncope in PE

- Reactions Phase
  *Goal: Allow the learner to process emotional and cognitive responses.*
  - “How did you feel about the case?”
  - “What went well? What did not go well?”
  - “What surprised you during the simulation?”
  - Description Phase
    *Goal: Establish a shared understanding of what happened.*
  - “In 2–3 sentences, please summarize this procedural case.”
  - “What helped or hindered your ability to perform the ultrasound?”
  - Analysis Phase
    *Goal: Facilitate reflection and provide structured feedback based on rubric scores.*
  - “What was your strategy for identifying the correct anatomical landmarks?”
  - “What would you do differently if you encountered this case again?”
  - For the parasternal short axis view: 12–15
    ○ To troubleshoot image acquisition, consider finding the parasternal long axis first and transitioning to the short axis image.
    ○ The parasternal short axis can be broken down into 4 distinct regions depending on structures viewed at the level of the aortic valve, mitral valve, papillary muscle or apex. The optimal view to assess for D-sign or septal flattening is at the level of the papillary muscles because this includes both ventricles in cross section without the mitral valve to obscure septal flattening.
  - For the inferior vena cava view:^7^,^14^–^16^
    ○ Consider using the curvilinear probe if the patient has an enlarged body habitus.
    ○ It is sometimes easier to find the traditional subxiphoid view and then rotate the probe to the sagittal plane.
    ○ Don’t mistake the aorta for the IVC because both may be larger vessels with pulsatility. The aorta tends to track posteriorly, or deep to the heart and lies more in the midline or patient’s left, while the IVC is in the patient’s right side and should have a clear junction with the hepatic veins and right atrium.
  - For the internal jugular view:^17^–^20^
    ○ If the internal jugular vein is entirely collapsed, as may be seen in healthy or volume-depleted patients, consider placing the patient in Trendelenburg position.
    ○ Consider light or minimal pressure because this may collapse the vein in healthy or volume-depleted patients.
  - Application/Summary Phase
  - “What is one key thing you learned today that you will apply the next time you perform an ultrasound?”

## References

[1] Beeson MS, Bhat R, Broder JS (2023). The 2022 Model of the clinical practice of emergency medicine. J Emerg Med.

[2] PoSaw LL, Wubben BM, Bertucci N, Bell GA, Healy H, Lee S (2021). Teaching emergency ultrasound to emergency medicine residents: a scoping review of structured training methods. JACEP Open [Internet].

[3] Johnson G, Reynard K (1994). Assessment of an objective structured clinical examination (OSCE) for undergraduate students in accident and emergency medicine. Emerg Med J.

[4] ABEM Certifying Exam [Internet].

[5] ABEM Certifying Exam Content [Internet].

[6] (2024). CE Sample Case: Ultrasound [Internet].

[7] Filopei J, Acquah SO, Bondarsky EE (2017). Diagnostic accuracy of point-of-care ultrasound performed by pulmonary critical care physicians for right ventricle assessment in patients with acute pulmonary embolism. Crit Care Med.

[8] Pivetta E, Moretto F, Masellis S (2022). Comparison between standard and ultrasound-integrated approach for risk stratification of syncope in the emergency department. Intern Emerg Med.

[9] Dwyer KH, Rempell JS, Stone MB (2018). Diagnosing centrally located pulmonary embolisms in the emergency department using point-of-care ultrasound. Am J Emerg Med.

[10] Taylor RA, Davis J, Liu R, Gupta V, Dziura J, Moore CL (2013). Point-of-care focused cardiac ultrasound for prediction of pulmonary embolism adverse outcomes. J Emerg Med.

[11] Hernandez J, Frallicciardi A, Nadir NA, Gothard MD, Ahmed RA (2020). Development of a Simulation Scenario Evaluation Tool (SSET): modified Delphi study. BMJ Simul Technol Enhanc Learn.

[12] Echocardiography for Emergency Physicians | Sonoguide [Internet].

[13] Jailyn (2021). 5 Min Sono – Right Heart Assessment - Core Ultrasound [Internet].

[14] Reardon RF, Laudenbach AP, Heller K, Ma OJ, Mateer JR, Reardon RF, Byars DV, Knapp BJ, Laudenbach AP (2021). Transthoracic Echocardiography [Internet]. Ma and Mateer’s Emergency Ultrasound.

[15] Kline JA, Tintinalli JE, Ma OJ, Yealy DM (2020). Venous thromboembolism including pulmonary embolism [Internet]. Tintinalli’s Emergency Medicine: A Comprehensive Study Guide.

[16] Avila J (2024). 5 Min Sono – IVC (2024) - Core Ultrasound [Internet].

[17] Kotwal A, Bryczkowski C, Mirza M Vascular Access | Sonoguide [Internet].

[18] Avila J (2020). Central Venous Access (2020 edition) - Core Ultrasound [Internet].

[19] Rose JS, Sylvester PJ, Abo AM, Ma OJ, Mateer JR, Reardon RF, Byars DV, Knapp BJ, Laudenbach AP (2021). Vascular Access [Internet]. Ma and Mateer’s Emergency Ultrasound.

[20] Wyatt C, Tintinalli JE, Ma OJ, Yealy DM (2020). Vascular access [Internet]. Tintinalli’s Emergency Medicine: A Comprehensive Study Guide.

